# Supplementary material for: Sheng Xue Ning as a Novel Agent that Promotes SCF-Driven Hematopoietic Stem/Progenitor Cell Proliferation to Promote Erythropoiesis
Source: Biomolecules. 2024 Sep 11;14(9):1147. doi: 10.3390/biom14091147 (PMC11429878; doi:10.3390/biom14091147)
Supplement: Supplementary file 1 [file biomolecules-14-01147-s001.zip › Supplementary.pdf]

Supplementary Data

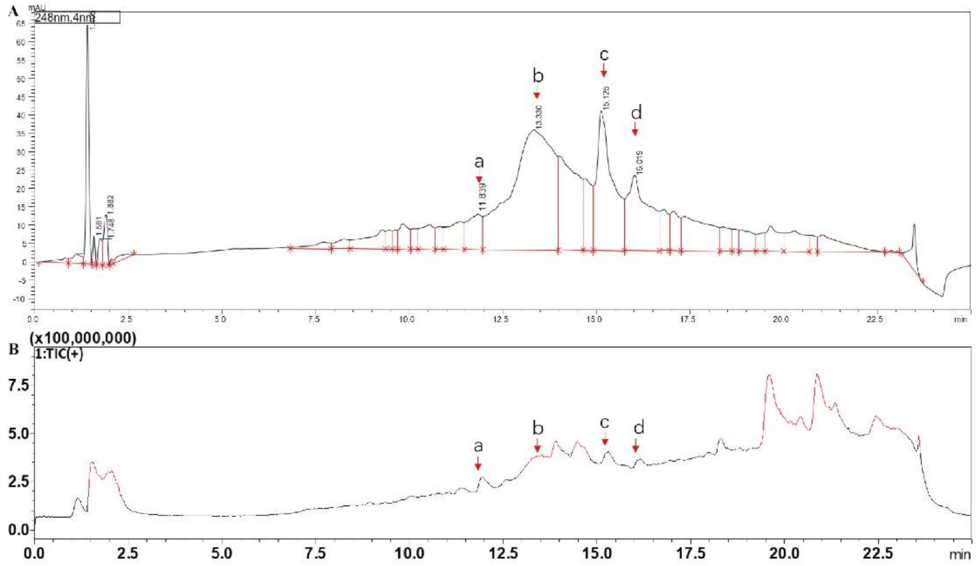

Figure S1. Liquid chromatography-tandem mass spectrometry (LC-MS/MS) analysis of SXN. Main structural formula: a.$C_{33}H_{32}N_4O_6Fe$ 596.45, b.$C_{33}H_{34}N_4O_4Fe$ 606.25, c.$C_{34}H_{34}N_4O_6Fe$ 650.20, d.$C_{33}H_{34}N_4O_4Fe$ 606.60. Instrumentation and Analysis Conditions: Samples were analyzed using an LC-MS-8040 UHPLC system (Shimadzu Corp., Kyoto, Japan). LabSolutions LC-MS ver.5.85 software was used for data acquisition and analysis. The following experimental parameters were set: Column: Shim; pack GIST column (2.1 mm ID.×50 mm l, 2.0 µm, P/N 227-30001-02); Flow rate: 0.45 m/min; Column temperature: 40 °C; Mobile phase: water with a small amount (0.05%) of ammonia water (solvent A) and acetonitrile with a small amount (0.05%) of ammonia water (solvent B); Sample tube temperature: -10 °C; Injection volume: 10 µL; Ionization mode: positive ion mode; Ion spray voltage: 3.5 kV; Source temperature: 400 °C; Desolvation temperature: 200° C; Collision energy for ZgI: 51 eV; Collision energy for IS: 52 eV; Atomizing gas flow rate: 2.5 L/min; Heating gas flow rate: 8.0 L/min; Drying gas flow rate: 10.0 L/min; Monitoring method: Multiple Reaction Monitoring (MRM) mode for precise monitoring of precursor ion to product ion transitions.

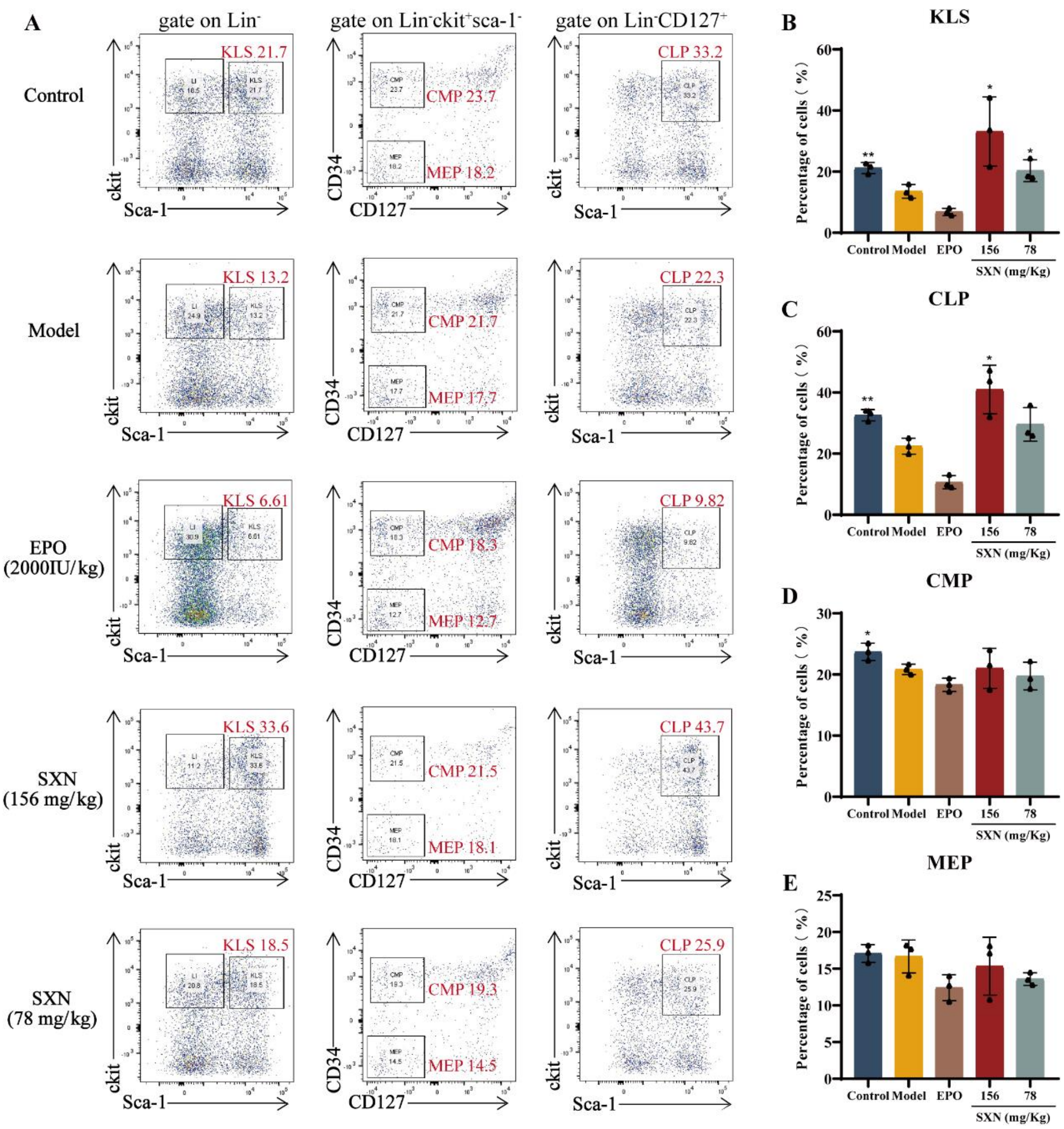

Figure S2. SXN promoted the proliferation of HSCs in X-ray irradiated mice in spleen. (**A**) The frequencies of HSCs (LSK, Lin$^-$ Sca-1$^+$ c-KIT$^+$), common lymphoid progenitor cell (CLP, Lin$^-$ CD127$^+$ Sca-1$^+$ c-KIT$^+$), common myeloid progenitor cell (CMP, Lin$^-$ CD127$^+$ Sca-1$^-$ c-KIT$^+$ CD34$^+$ CD16/32$^-$), and megakaryocyte-erythroid progenitor cell (MEP, Lin$^-$ CD127$^+$ Sca-1$^-$ c-KIT$^+$ CD34$^-$ CD16/32$^-$) in spleen of each group (n = 3). (**B-E**) The histogram represents the percentage of KLS, CLP, CMP, and MEP cells in each group. Data represent the mean ± SD of three independent experiments. * $p < 0.05$, ** $p < 0.01$, *** $p < 0.001$ vs. the model group.

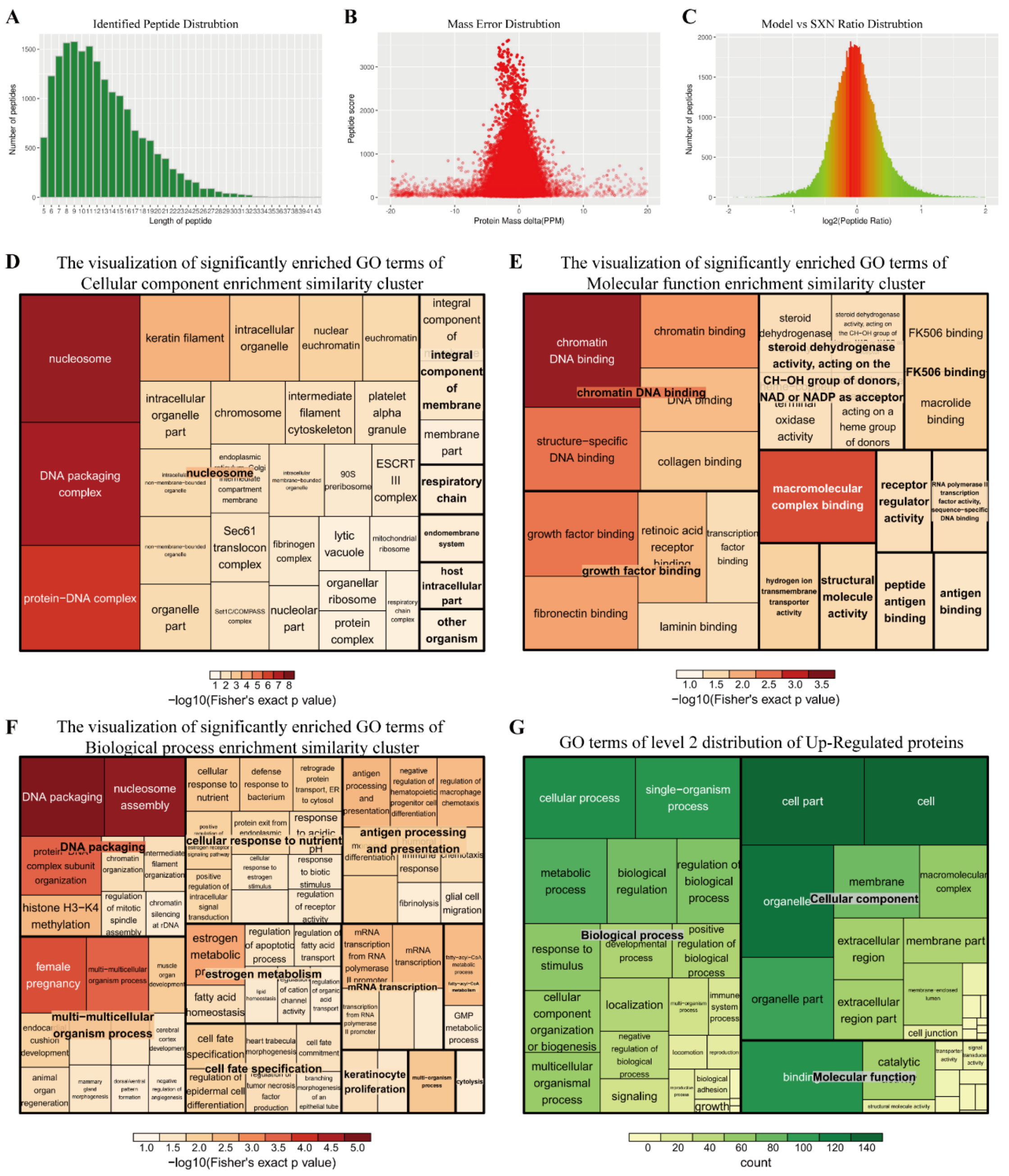

Figure S3. iTraq Quantitative Proteomics analysis. **(A)** Quality control of identified peptide distribution. **(B)** Mass error distribution. **(C)** Ratio distrubtion between the model group and the SXN treatment group. **(D)** The visualization of significantly enriched GO terms of cellular component enrichment similarity cluster. **(E)** The visualization of significantly enriched GO terms of molecular function enrichment similarity cluster. **(F)** The visualization of significantly enriched GO terms of biological process enrichment similarity cluster. **(G)** GO terms of level 2 distribution

of Up-Regulated proteins.

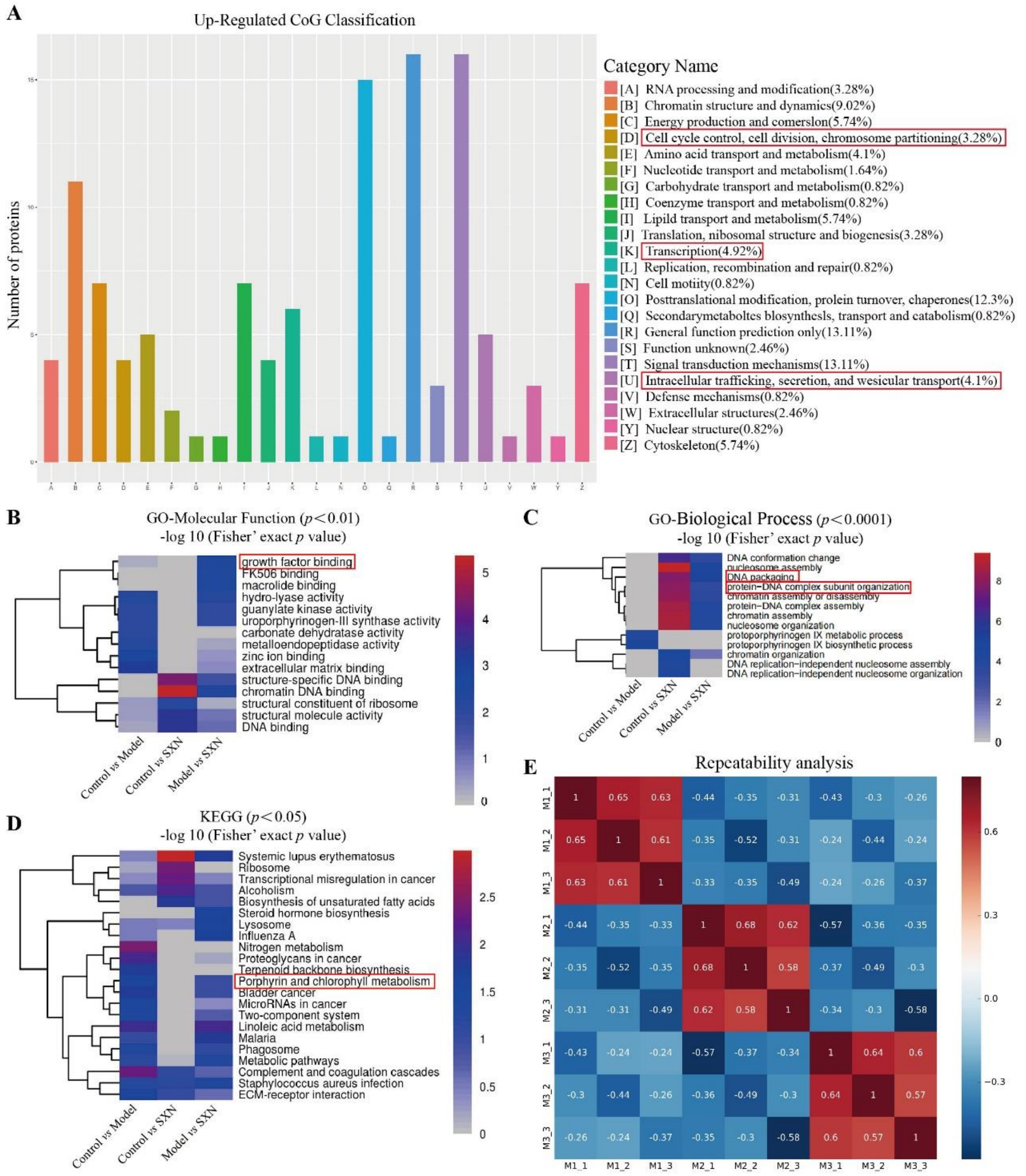

Figure S4. iTraq Quantitative Proteomics analysis. **(A)** Up-regulated CoG classification between the model group and the SXN treatment group. **(B)** GO molecular functional function cluster. **(C)** GO biological process function cluster. **(D)** KEGG Pathway function cluster. **(E)** Repeatability analysis.
