# Supplementary material for: Sheng Xue Ning as a Novel Agent that Promotes SCF-Driven Hematopoietic Stem/Progenitor Cell Proliferation to Promote Erythropoiesis
Source: Biomolecules. 2024 Sep 11;14(9):1147. doi: 10.3390/biom14091147 (PMC11429878; doi:10.3390/biom14091147)
Supplement: Supplementary file 1 [file biomolecules-14-01147-s001.zip › WB images.pdf]

<table>
<tr><th colspan="6">Immunoblot images of SCF in adipocytes differentiated MSCs treated with SXN (2.5, 5, and 10 μg/mL)</th></tr>
<tr><th>Name of protein</th><th>WB figures</th><th>Group</th><th>IntDen</th><th></th><th></th></tr>
<tr><td rowspan="12">SCF</td><td rowspan="4">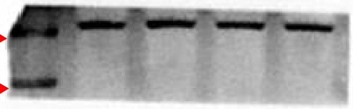</td><td>control</td><td>96991</td><td></td><td></td></tr>
<tr><td>SXN-2.5</td><td>85563</td><td></td><td></td></tr>
<tr><td>SXN-5</td><td>77564</td><td></td><td></td></tr>
<tr><td>SXN-10</td><td>75267</td><td></td><td></td></tr>
<tr><td rowspan="4">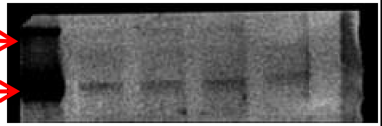</td><td>control</td><td>50823</td><td></td><td></td></tr>
<tr><td>SXN-2.5</td><td>58447</td><td></td><td></td></tr>
<tr><td>SXN-5</td><td>70161</td><td></td><td></td></tr>
<tr><td>SXN-10</td><td>54422</td><td></td><td></td></tr>
<tr><td rowspan="4">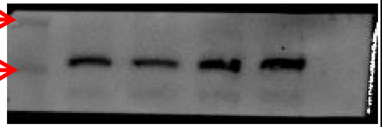</td><td>control</td><td>221526</td><td></td><td></td></tr>
<tr><td>SXN-2.5</td><td>196768</td><td></td><td></td></tr>
<tr><td>SXN-5</td><td>262119</td><td></td><td></td></tr>
<tr><td>SXN-10</td><td>287892</td><td></td><td></td></tr>
<tr><th>Name of protein</th><th>WB figures</th><th>Group</th><th>IntDen</th><th>intensity ratio</th><th>normalization</th></tr>
<tr><td rowspan="12">GAPDH</td><td rowspan="4">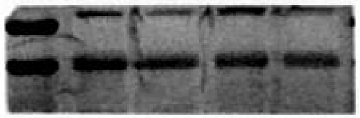</td><td>control</td><td>80138</td><td>1.210299733</td><td>1</td></tr>
<tr><td>SXN-2.5</td><td>66304</td><td>1.29046513</td><td>1.066235987</td></tr>
<tr><td>SXN-5</td><td>54962</td><td>1.411229577</td><td>1.166016598</td></tr>
<tr><td>SXN-10</td><td>55355</td><td>1.35971457</td><td>1.123452755</td></tr>
<tr><td rowspan="4">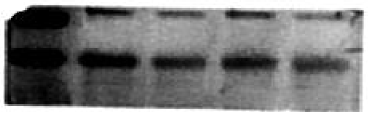</td><td>control</td><td>108414</td><td>0.468786319</td><td>1</td></tr>
<tr><td>SXN-2.5</td><td>93600</td><td>0.624433761</td><td>1.33202215</td></tr>
<tr><td>SXN-5</td><td>109396</td><td>0.641348861</td><td>1.368104902</td></tr>
<tr><td>SXN-10</td><td>106987</td><td>0.508678625</td><td>1.085096991</td></tr>
<tr><td rowspan="4">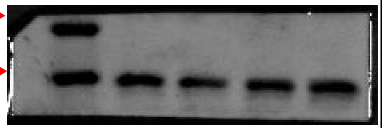</td><td>control</td><td>243395</td><td>0.910150167</td><td>1</td></tr>
<tr><td>SXN-2.5</td><td>204984</td><td>0.959918823</td><td>1.054681807</td></tr>
<tr><td>SXN-5</td><td>230480</td><td>1.137274384</td><td>1.249545871</td></tr>
<tr><td>SXN-10</td><td>268912</td><td>1.07058071</td><td>1.176268212</td></tr>
</table>

Immunoblot images of perilipin-1 in adipocytes differentiated MSCs treated with SXN (2.5, 5, and 10 µg/mL)					
Name of protein	WB figures	Group	IntDen		
perilipin-1	control 2.5 5 10; 70, 50 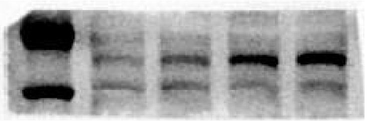	control	38781		
		SXN-2.5	42953		
		SXN-5	63205		
		SXN-10	90629		
	70, 50 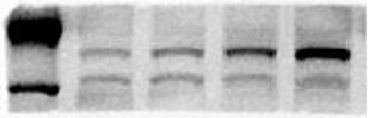	control	70161		
		SXN-2.5	78270		
		SXN-5	93458		
		SXN-10	99415		
	70, 50 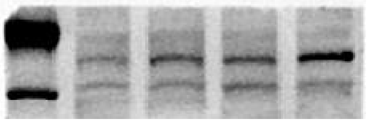	control	60421		
		SXN-2.5	79820		
		SXN-5	75103		
		SXN-10	85157		
Name of protein	WB figures	Group	IntDen	intensity ratio	normalization
ACTIN	control 2.5 5 10; 40, 35 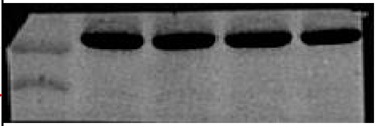	control	135255	0.286725075	1
		SXN-2.5	157853	0.272107594	0.949019174
		SXN-5	173082	0.365173733	1.273602362
		SXN-10	153992	0.588530573	2.052595412
	40, 35 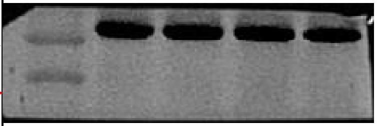	control	108414	0.647158116	1
		SXN-2.5	93600	0.836217949	1.292138548
		SXN-5	109396	0.854309116	1.320093335
		SXN-10	106987	0.929225046	1.435854736
	50, 40 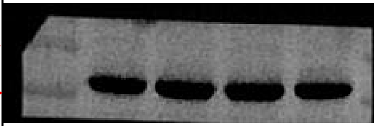	control	142553	0.423849375	1
		SXN-2.5	114726	0.695744644	1.641490313
		SXN-5	118414	0.634240884	1.496382726
		SXN-10	115562	0.736894481	1.738576305

Immunoblot images of SCF after treatment with SXN (2.5, 5, and 10 μg/mL) in MSCs for 3 days.

Name of protein	WB figures	Group	IntDen
SCF	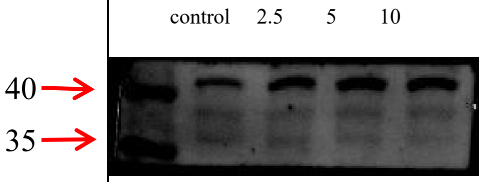	control	69900
		SXN-2.5	130579
		SXN-5	143965
		SXN-10	130254
	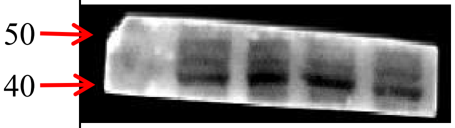	control	59313
		SXN-2.5	74184
		SXN-5	77789
		SXN-10	65103
	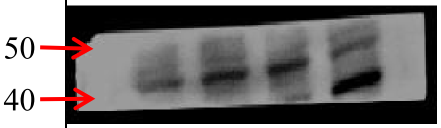	control	25287
		SXN-2.5	42445
		SXN-5	33796
		SXN-10	32089

Name of protein	WB figures	Group	IntDen	intensity ratio	normalization
GAPDH	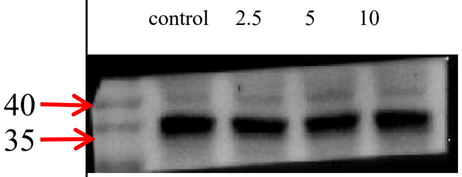	control	368676	0.18959737	1
		SXN-2.5	354330	0.368523693	1.943717323
		SXN-5	371761	0.387251487	2.042493979
		SXN-10	365398	0.356471573	1.880150412
	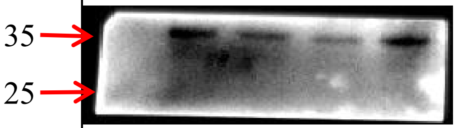	control	94471	0.627843465	1
		SXN-2.5	71941	1.031178327	1.642413092
		SXN-5	53926	1.442513815	2.297569211
		SXN-10	70442	0.924207149	1.472034353
	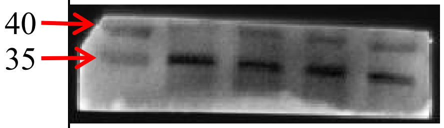	control	50597	0.499772714	1
		SXN-2.5	48789	0.86997069	1.740732669
		SXN-5	50301	0.671875311	1.344361731
		SXN-10	35170	0.912396929	1.825623737

Immunoblot images of PI3K/AKT signaling pathways after treatment with SXN (2.5, 5, and 10 μg/mL) in MSCs for 3 days.

Name of protein	WB figures	Group	IntDen
P-PI3K	control 2.5 5 10 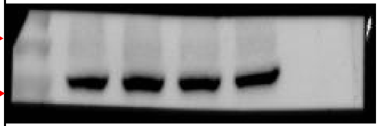	control	151358
		SXN-2.5	186060
		SXN-5	168475
		SXN-10	181247
	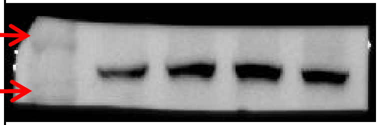	control	111791
		SXN-2.5	173088
		SXN-5	211094
		SXN-10	179216
	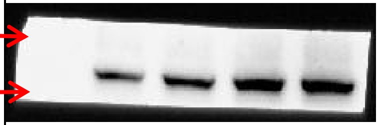	control	90118
		SXN-2.5	130709
		SXN-5	189512
		SXN-10	216820

Name of protein	WB figures	Group	IntDen	intensity ratio	normalization
PI3K	control 2.5 5 10 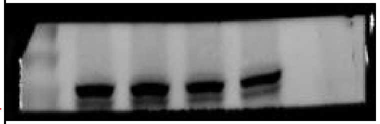	control	202862	0.746113121	1
		SXN-2.5	213580	0.871148984	1.167582983
		SXN-5	192687	0.874345441	1.171867129
		SXN-10	187890	0.964644207	1.292892699
	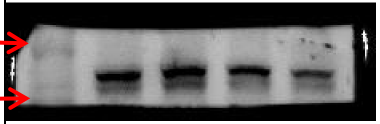	control	196817	0.567994635	1
		SXN-2.5	218971	0.790460837	1.39166955
		SXN-5	166357	1.268921656	2.234038104
		SXN-10	133272	1.34473858	2.367519863
	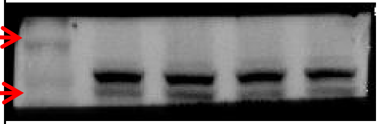	control	179508	0.502027765	1
		SXN-2.5	174712	0.748139796	1.490235896
		SXN-5	161775	1.17145418	2.333445005
		SXN-10	151741	1.428882108	2.846221282

Immunoblot images of PI3K/AKT signaling pathways after treatment with SXN (2.5, 5, and 10 µg/mL) in MSCs for 3 days.

Name of protein	WB figures	Group	IntDen
P-AKT	control 2.5 5 10 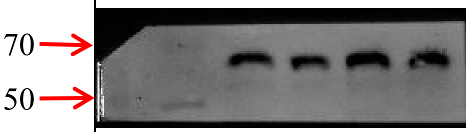	control	237899
		SXN-2.5	215636
		SXN-5	319318
		SXN-10	283878
	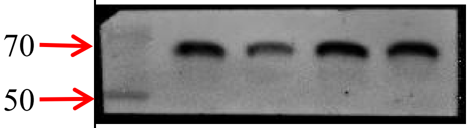	control	222538
		SXN-2.5	144910
		SXN-5	259679
		SXN-10	248183
	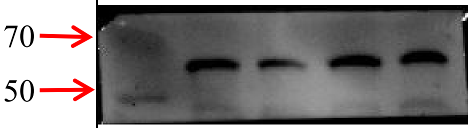	control	150068
		SXN-2.5	107990
		SXN-5	176731
		SXN-10	176929

Name of protein	WB figures	Group	IntDen	intensity ratio	normalization
AKT	control 2.5 5 10 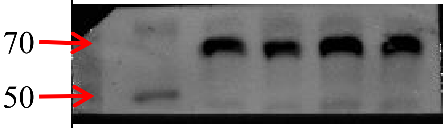	control	318094	0.747888989	1
		SXN-2.5	257077	0.83879927	1.12155585
		SXN-5	368782	0.865871979	1.157754682
		SXN-10	360209	0.788092469	1.053755946
	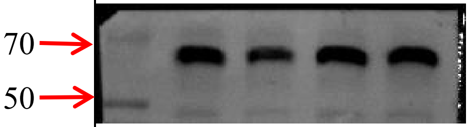	control	255047	0.872537219	1
		SXN-2.5	157097	0.922423725	1.057174073
		SXN-5	260144	0.998212528	1.144034326
		SXN-10	255327	0.972020194	1.114015738
	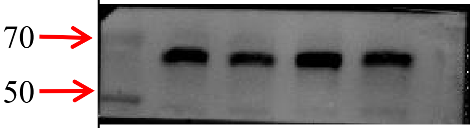	control	330838	0.453599647	1
		SXN-2.5	256647	0.420772501	0.927629692
		SXN-5	371036	0.476317662	1.050083846
		SXN-10	349662	0.50600008	1.115521327

Immunoblot images of SCF/C-KIT signaling pathways after treatment with SXN (2.5, 5, and 10 μg/mL) in BMNCs for 6 days.

Name of protein	WB figures	Group	IntDen		
SCF	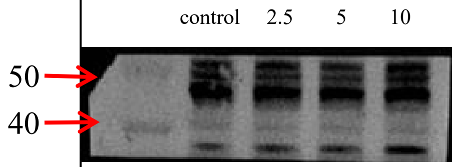	control	71296		
		SXN-2.5	73785		
		SXN-5	70680		
		SXN-10	76150		
	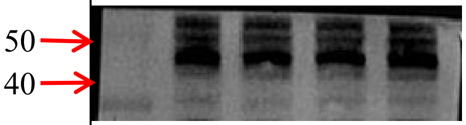	control	87887		
		SXN-2.5	88003		
		SXN-5	89142		
		SXN-10	100536		
	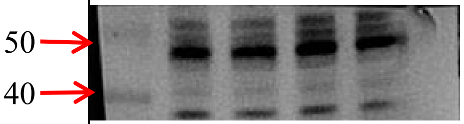	control	82794		
		SXN-2.5	89316		
		SXN-5	91352		
		SXN-10	94550		
Name of protein	WB figures	Group	IntDen	intensity ratio	normalization
GAPDH	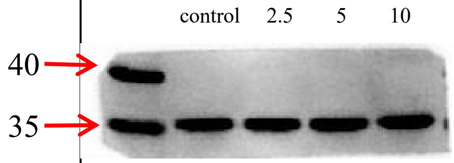	control	84625	0.842493353	1
		SXN-2.5	84014	0.878246483	1.042437284
		SXN-5	85975	0.822099448	0.975793393
		SXN-10	84792	0.898080008	1.065978745
	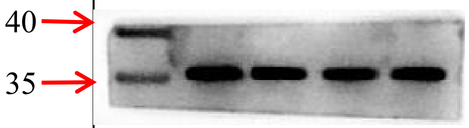	control	85793	1.024407586	1
		SXN-2.5	74261	1.185050026	1.156814966
		SXN-5	82170	1.084848485	1.059000831
		SXN-10	86094	1.167746881	1.139924086
	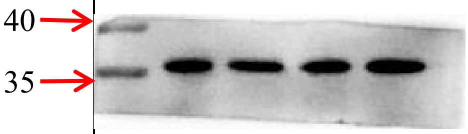	control	97495	0.84921278	1
		SXN-2.5	87469	1.021116053	1.20242662
		SXN-5	89641	1.019087248	1.200037578
		SXN-10	94871	0.996616458	1.173576848

Immunoblot images of SCF/C-KIT signaling pathways after treatment with SXN (2.5, 5, and 10 μg/mL) in BMNCs for 6 days.

Name of protein	WB figures	Group	IntDen
C-KIT	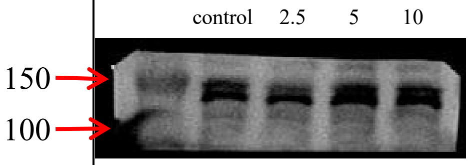	control	130160
		SXN-2.5	124278
		SXN-5	148746
		SXN-10	142365
	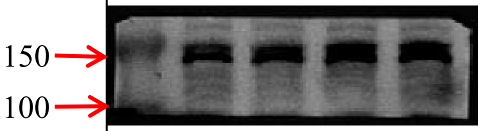	control	116942
		SXN-2.5	140174
		SXN-5	150267
		SXN-10	151581
	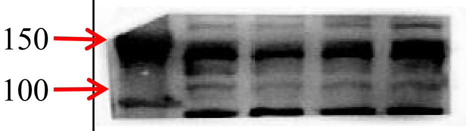	control	132555
		SXN-2.5	126899
		SXN-5	164177
		SXN-10	229753

Name of protein	WB figures	Group	IntDen	intensity ratio	normalization
GAPDH	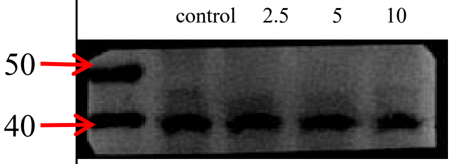	control	65964	1.973197502	1
		SXN-2.5	71321	1.742516229	0.883092659
		SXN-5	66049	2.252055292	1.141322797
		SXN-10	49702	2.864371655	1.451639612
	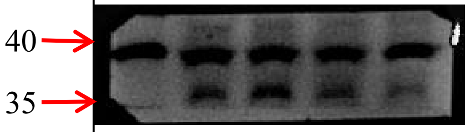	control	91946	1.271855219	1
		SXN-2.5	91193	1.537113594	1.208560197
		SXN-5	90766	1.655542824	1.301675536
		SXN-10	91652	1.653875529	1.30036462
	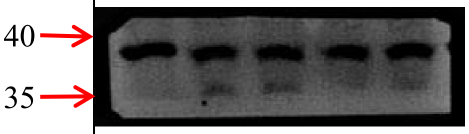	control	60163	2.203264465	1
		SXN-2.5	61310	2.069792856	0.939420977
		SXN-5	54812	2.995274757	1.359471278
		SXN-10	60951	3.769470558	1.710857057

Immunoblot images of PI3K/AKT signaling pathways after treatment with SXN (2.5, 5, and 10 μg/mL) in BMNCs for 6 days.

Name of protein	WB figures	Group	IntDen
100 → 70 →	control 2.5 5 10 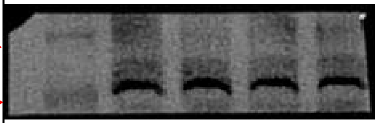	control	89208
		SXN-2.5	91787
		SXN-5	83185
		SXN-10	81475
P-PI3K 100 → 70 →	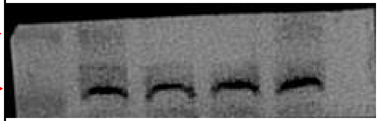	control	52503
		SXN-2.5	51324
		SXN-5	60046
		SXN-10	60597
100 → 70 →	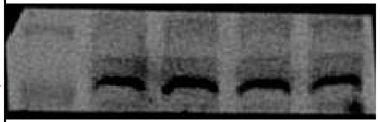	control	80365
		SXN-2.5	92433
		SXN-5	76835
		SXN-10	76930

Name of protein	WB figures	Group	IntDen	intensity ratio	normalization
100 → 70 →	control 2.5 5 10 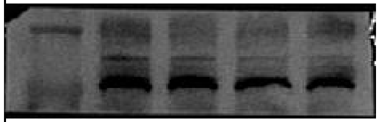	control	108947	0.81882016	1
		SXN-2.5	100893	0.909745969	1.111044907
		SXN-5	83335	0.998200036	1.219071152
		SXN-10	84463	0.964623563	1.178065233
PI3K 100 → 70 →	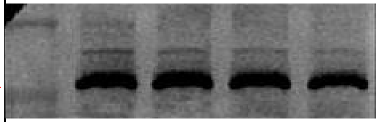	control	99057	0.530028166	1
		SXN-2.5	96630	0.531139398	1.002096553
		SXN-5	91591	0.655588431	1.236893572
		SXN-10	86581	0.699887966	1.320473159
100 → 70 →	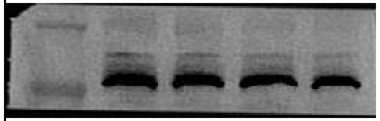	control	89342	0.899520942	1
		SXN-2.5	82599	1.119057131	1.244059009
		SXN-5	75883	1.012545629	1.125649868
		SXN-10	64834	1.186568776	1.319111896

Immunoblot images of PI3K/AKT signaling pathways after treatment with SXN (2.5, 5, and 10 µg/mL) in BMNCs for 6 days.

Name of protein	WB figures	Group	IntDen
P-AKT	control 2.5 5 10<br>70 → 50 → 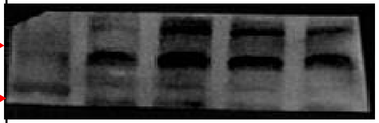	control	80803
		SXN-2.5	111146
		SXN-5	93445
		SXN-10	81221
	70 → 50 → 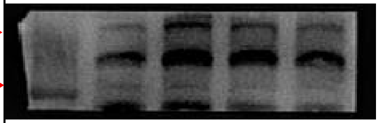	control	72932
		SXN-2.5	111536
		SXN-5	99611
		SXN-10	93368
	70 → 50 → 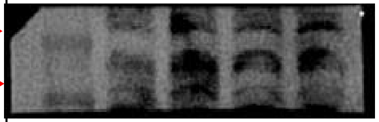	control	61602
		SXN-2.5	111913
		SXN-5	83814
		SXN-10	95502

Name of protein	WB figures	Group	IntDen	intensity ratio	normalization
AKT	control 2.5 5 10<br>70 → 50 → 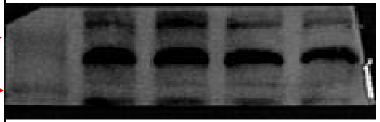	control	87813	0.920171273	1
		SXN-2.5	90843	1.223495481	1.329638859
		SXN-5	78470	1.190837263	1.294147402
		SXN-10	65693	1.236372216	1.343632705
	70 → 50 → 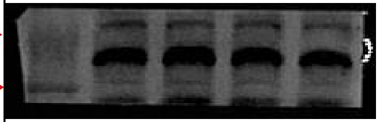	control	107177	0.68048182	1
		SXN-2.5	116447	0.957826307	1.407570753
		SXN-5	100819	0.988018132	1.451939057
		SXN-10	87709	1.064520175	1.564362403
	70 → 50 → 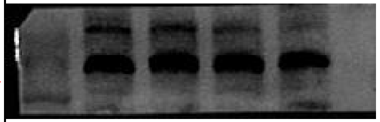	control	107046	0.575472227	1
		SXN-2.5	112720	0.992840667	1.725262525
		SXN-5	110025	0.761772324	1.323734298
		SXN-10	94610	1.009428179	1.754086699

Immunoblot images of SCF/C-KIT in spleen cells of each group mice.					
Name of protein	WB figures	Group	IntDen		
SCF	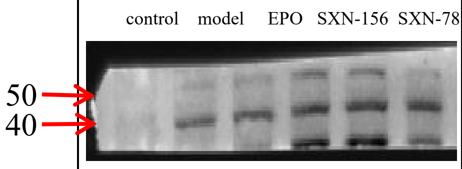	control	24793		
		model	24216		
		EPO	29351		
		SXN-156	33466		
		SXN-78	32102		
	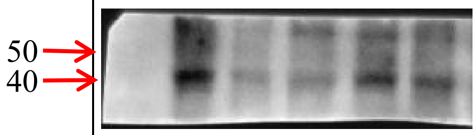	control	94707		
		model	70911		
		EPO	59004		
		SXN-156	74199		
		SXN-78	76188		
	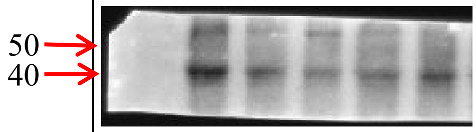	control	83523		
		model	30207		
		EPO	43966		
		SXN-156	68280		
		SXN-78	63857		
Name of protein	WB figures	Group	IntDen	intensity ratio	normalization
GAPDH	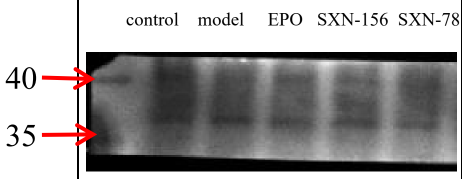	control	62356	0.39760408	1
		model	78343	0.309102281	0.777412247
		EPO	76860	0.381876138	0.960443209
		SXN-156	78172	0.428107251	1.076717449
		SXN-78	67869	0.472999455	1.189624249
	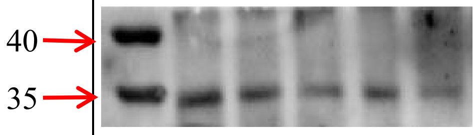	control	26719	3.544556308	1
		model	27702	2.559779077	0.722171932
		EPO	24091	2.449213399	0.690978838
		SXN-156	25588	2.899757699	0.818087638
		SXN-78	20880	3.648850575	1.029423786
	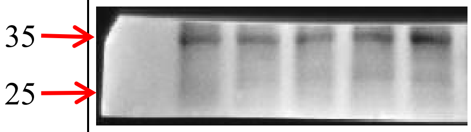	control	44923	1.859248047	1
		model	30243	0.998809642	0.537211613
		EPO	32151	1.367484682	0.735504165
		SXN-156	34865	1.958411014	1.053334985
		SXN-78	42505	1.502340901	0.808036832

Immunoblot images of SCF/C-KIT in spleen cells of each group mice.					
Name of protein	WB figures	Group	IntDen		
C-KIT	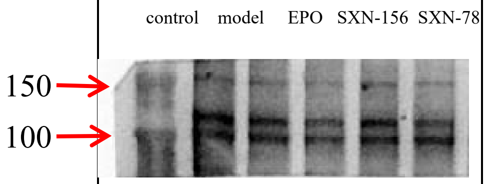	control	146727		
		model	122410		
		EPO	112345		
		SXN-156	128325		
		SXN-78	121325		
	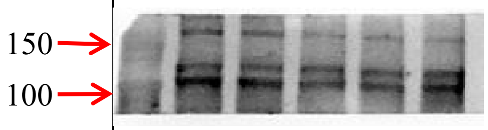	control	110247		
		model	97229		
		EPO	101293		
		SXN-156	178713		
		SXN-78	118147		
	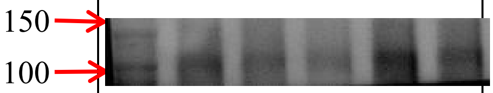	control	53258		
		model	39971		
		EPO	27894		
		SXN-156	77130		
		SXN-78	114869		
Name of protein	WB figures	Group	IntDen	intensity ratio	normalization
GAPDH	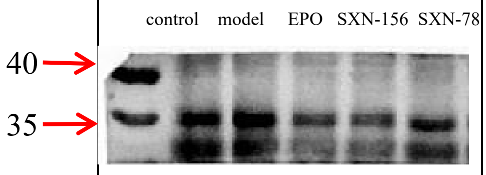	control	108738	1.349362688	1
		model	117691	1.040096524	0.770805754
		EPO	81327	1.381398552	1.023741477
		SXN-156	75138	1.707857542	1.265677165
		SXN-78	88660	1.368429957	1.014130574
	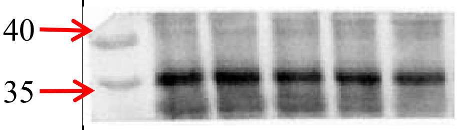	control	116114	0.949472071	1
		model	129039	0.75348538	0.793583512
		EPO	117313	0.863442244	0.909391936
		SXN-156	108456	1.647792653	1.735483017
		SXN-78	103751	1.138755289	1.199356279
	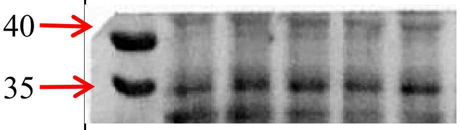	control	81479	0.653640815	1
		model	85166	0.469330484	0.718025057
		EPO	76847	0.362980988	0.555321791
		SXN-156	65900	1.170409712	1.790600715
		SXN-78	76010	1.511235364	2.31202723

Immunoblot images of PI3K/AKT in spleen cells of each group mice.					
Name of protein	WB figures	Group	IntDen		
P-PI3K	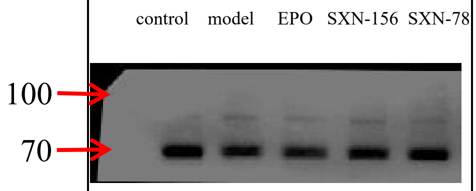	control	28690		
		model	15016		
		EPO	16978		
		SXN-156	28599		
		SXN-78	25432		
	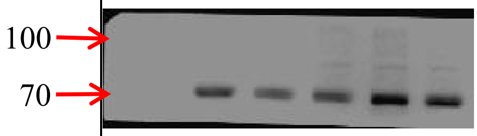	control	57772		
		model	44731		
		EPO	48325		
		SXN-156	78774		
		SXN-78	67311		
	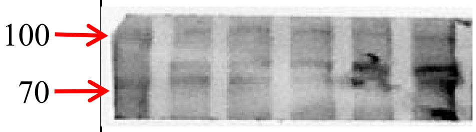	control	45690		
		model	40958		
		EPO	34557		
		SXN-156	85676		
		SXN-78	120013		
Name of protein	WB figures	Group	IntDen	intensity ratio	normalization
PI3K	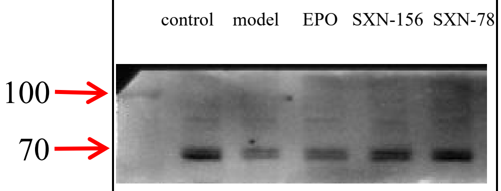	control	100613	0.285152018	1
		model	68411	0.219496865	0.769753853
		EPO	67505	0.251507296	0.882011277
		SXN-156	71192	0.401716485	1.408780087
		SXN-78	80362	0.316467982	1.109821998
	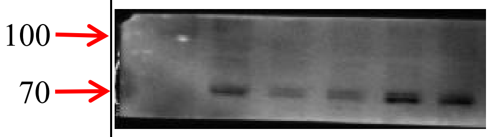	control	30683	1.882866734	1
		model	21719	2.059533128	1.093828411
		EPO	18669	2.588515721	1.374773729
		SXN-156	28115	2.801849547	1.488076397
		SXN-78	29879	2.252786238	1.19646611
	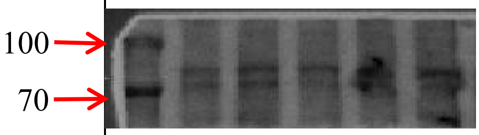	control	26496	1.724411232	1
		model	25027	1.636552523	0.949050025
		EPO	23592	1.464776195	0.849435545
		SXN-156	29695	2.885199529	1.673150508
		SXN-78	29326	4.092375367	2.373201526

Immunoblot images of PI3K/AKT in spleen cells of each group mice.

Name of protein	WB figures	Group	IntDen
P-AKT	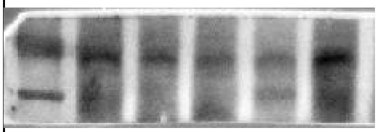	control	115120
		model	96274
		EPO	93440
		SXN-156	88469
		SXN-78	121298
	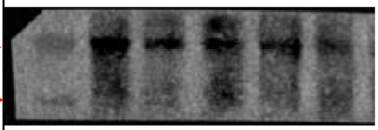	control	117104
		model	80128
		EPO	100069
		SXN-156	94387
		SXN-78	69397
	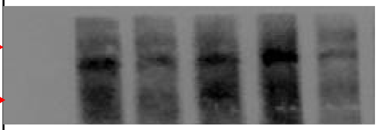	control	115495
		model	77644
		EPO	116557
		SXN-156	135097
		SXN-78	55103

Name of protein	WB figures	Group	IntDen	intensity ratio	normalization
AKT	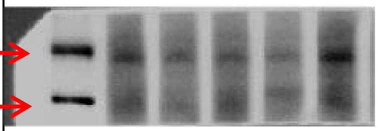	control	56382	2.041786386	1
		model	52119	1.84719584	0.904695934
		EPO	47028	1.98690142	0.973119144
		SXN-156	45148	1.959533091	0.959715034
		SXN-78	39771	3.049910739	1.493746241
	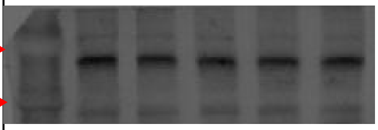	control	51765	2.26222351	1
		model	43632	1.836450312	0.811789951
		EPO	44798	2.233782758	0.987427966
		SXN-156	43201	2.184833684	0.965790372
		SXN-78	48713	1.424609447	0.629738592
	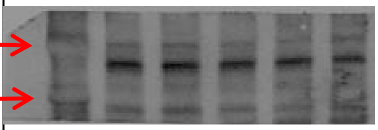	control	62329	1.85298978	1
		model	56267	1.379920735	0.744699593
		EPO	55455	2.101830313	1.134291368
		SXN-156	73147	1.846924686	0.99672686
		SXN-78	91575	0.601725362	0.324732154

Immunoblot images of PI3K/AKT in bone marrow cells of each group mice.

Name of protein	WB figures	Group	IntDen
P-PI3K	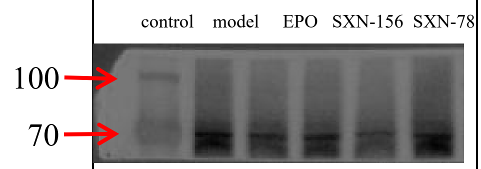	control	71407
		model	54404
		EPO	59824
		SXN-156	54439
		SXN-78	62914
	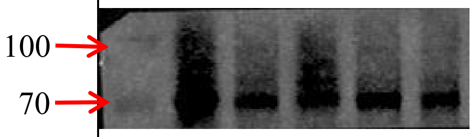	control	144742
		model	86010
		EPO	88171
		SXN-156	103866
		SXN-78	88833
	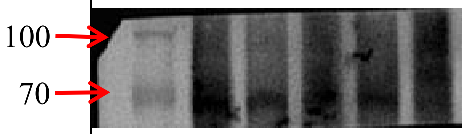	control	109903
		model	90634
		EPO	107117
		SXN-156	106813
		SXN-78	97596

Name of protein	WB figures	Group	IntDen	intensity ratio	normalization
PI3K	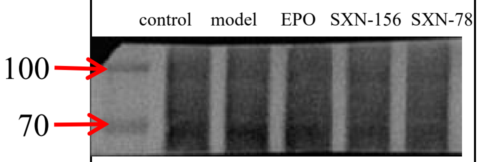	control	83233	0.857916932	1
		model	99822	0.545010118	0.635271432
		EPO	104962	0.569958652	0.664351793
		SXN-156	86038	0.632732049	0.737521344
		SXN-78	82138	0.765954856	0.892807716
	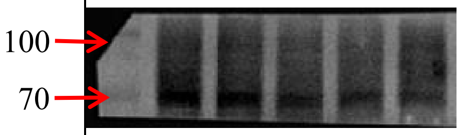	control	115050	1.258079096	1
		model	111847	0.768996933	0.611246889
		EPO	91308	0.965643755	0.76755409
		SXN-156	88185	1.177819357	0.936204537
		SXN-78	85811	1.03521693	0.822855203
	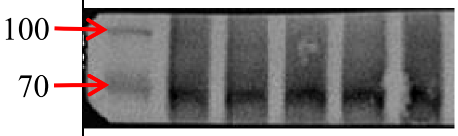	control	87577	1.254929947	1
		model	88632	1.022587779	0.814856463
		EPO	98194	1.090871133	0.869268548
		SXN-156	93139	1.146812828	0.913846092
		SXN-78	83348	1.170945914	0.933076716

Immunoblot images of PI3K/AKT in bone marrow cells of each group mice.

Name of protein	WB figures	Group	IntDen	intensity ratio	normalization
	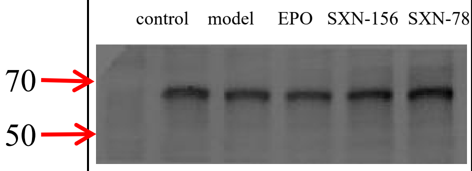	control	130264		
		model	118555		
		EPO	148528		
		SXN-156	144952		
		SXN-78	131553		
	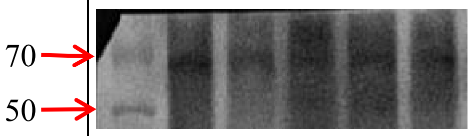	control	60810		
		model	53574		
P-AKT		EPO	54673		
		SXN-156	68484		
		SXN-78	83883		
	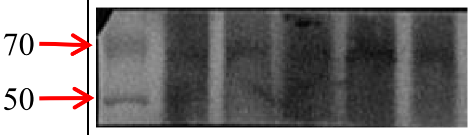	control	79483		
		model	81092		
		EPO	107245		
		SXN-156	109540		
		SXN-78	88639		
Name of protein	WB figures	Group	IntDen	intensity ratio	normalization
	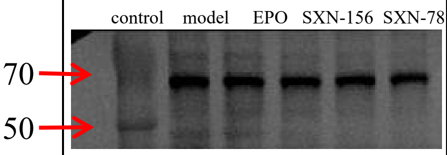	control	89052	1.462785788	1
		model	84698	1.399737892	0.95689875
		EPO	79478	1.86879388	1.277558133
		SXN-156	89427	1.620897492	1.108089445
		SXN-78	93228	1.411088943	0.964658636
	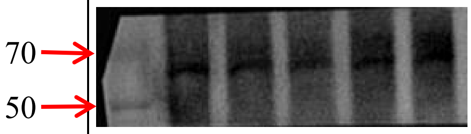	control	56648	1.073471261	1
		model	53360	1.004010495	0.935293315
AKT		EPO	47548	1.149848574	1.071149844
		SXN-156	45319	1.511154262	1.407726799
		SXN-78	39658	2.115159615	1.9703924
	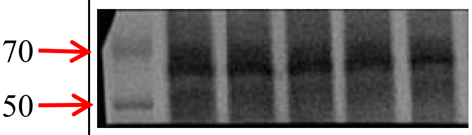	control	97029	0.819167465	1
		model	101896	0.795831043	0.971512025
		EPO	104136	1.029855189	1.257197378
		SXN-156	102173	1.072103198	1.308771702
		SXN-78	94923	0.933798974	1.139936598

<table>
<tr><th colspan="6">Immunoblot images of SCF/c-KIT in bone marrow cells of each group mice.</th></tr>
<tr><th>Name of protein</th><th>WB figures</th><th>Group</th><th>IntDen</th><th></th><th></th></tr>
<tr><td rowspan="15">SCF</td><td rowspan="5">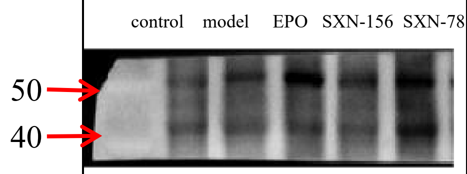</td><td>control</td><td>46474</td><td></td><td></td></tr>
<tr><td>model</td><td>45163</td><td></td><td></td></tr>
<tr><td>EPO</td><td>52317</td><td></td><td></td></tr>
<tr><td>SXN-156</td><td>47499</td><td></td><td></td></tr>
<tr><td>SXN-78</td><td>70614</td><td></td><td></td></tr>
<tr><td rowspan="5">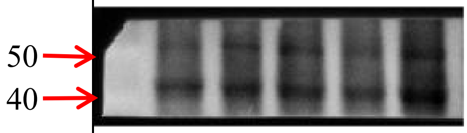</td><td>control</td><td>71955</td><td></td><td></td></tr>
<tr><td>model</td><td>72900</td><td></td><td></td></tr>
<tr><td>EPO</td><td>84001</td><td></td><td></td></tr>
<tr><td>SXN-156</td><td>67150</td><td></td><td></td></tr>
<tr><td>SXN-78</td><td>90762</td><td></td><td></td></tr>
<tr><td rowspan="5">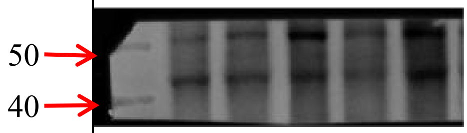</td><td>control</td><td>46607</td><td></td><td></td></tr>
<tr><td>model</td><td>48586</td><td></td><td></td></tr>
<tr><td>EPO</td><td>60375</td><td></td><td></td></tr>
<tr><td>SXN-156</td><td>51588</td><td></td><td></td></tr>
<tr><td>SXN-78</td><td>78025</td><td></td><td></td></tr>
<tr><th>Name of protein</th><th>WB figures</th><th>Group</th><th>IntDen</th><th>intensity ratio</th><th>normalization</th></tr>
<tr><td rowspan="15">GAPDH</td><td rowspan="5">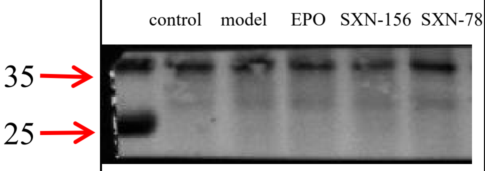</td><td>control</td><td>47661</td><td>0.975094941</td><td>1</td></tr>
<tr><td>model</td><td>49148</td><td>0.918918369</td><td>0.942388613</td></tr>
<tr><td>EPO</td><td>48897</td><td>1.069942941</td><td>1.097270528</td></tr>
<tr><td>SXN-156</td><td>43540</td><td>1.090927882</td><td>1.118791449</td></tr>
<tr><td>SXN-78</td><td>51089</td><td>1.382176202</td><td>1.41747859</td></tr>
<tr><td rowspan="5">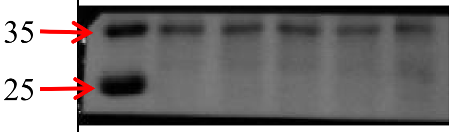</td><td>control</td><td>27673</td><td>2.600187909</td><td>1</td></tr>
<tr><td>model</td><td>28796</td><td>2.531601611</td><td>0.973622561</td></tr>
<tr><td>EPO</td><td>30806</td><td>2.726774005</td><td>1.048683442</td></tr>
<tr><td>SXN-156</td><td>24296</td><td>2.763829437</td><td>1.062934501</td></tr>
<tr><td>SXN-78</td><td>25174</td><td>3.60538651</td><td>1.386586907</td></tr>
<tr><td rowspan="5">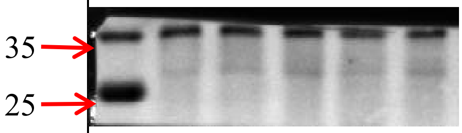</td><td>control</td><td>44319</td><td>1.051625714</td><td>1</td></tr>
<tr><td>model</td><td>50369</td><td>0.964601243</td><td>0.917247677</td></tr>
<tr><td>EPO</td><td>49218</td><td>1.226685359</td><td>1.166465733</td></tr>
<tr><td>SXN-156</td><td>44471</td><td>1.160036878</td><td>1.103089115</td></tr>
<tr><td>SXN-78</td><td>42993</td><td>1.814830321</td><td>1.725737872</td></tr>
</table>

Immunoblot images of SCF/c-KIT in bone marrow cells of each group mice.

Name of protein	WB figures	Group	IntDen
C-KIT	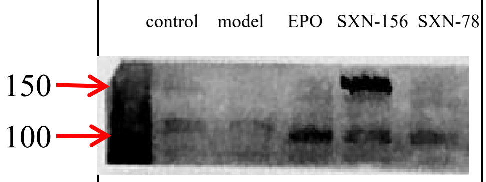	control	80113
		model	79687
		EPO	102957
		SXN-156	101938
		SXN-78	90175
	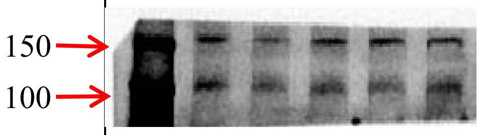	control	88540
		model	79876
		EPO	79957
		SXN-156	79474
		SXN-78	93938
	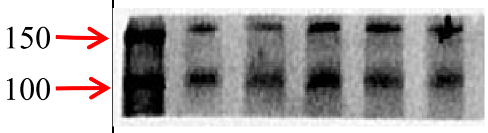	control	99970
		model	98607
		EPO	116230
		SXN-156	107069
		SXN-78	100257

Name of protein	WB figures	Group	IntDen	intensity ratio	normalization
ACTIN	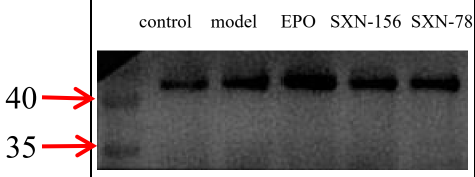	control	118784	0.674442686	1
		model	128064	0.622243566	0.922604068
		EPO	146887	0.700926563	1.039267794
		SXN-156	132049	0.771971011	1.144605801
		SXN-78	130022	0.693536478	1.028310475
	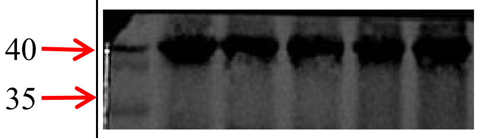	control	126938	0.697505869	1
		model	119610	0.667803695	0.957416597
		EPO	113270	0.705897413	1.012030787
		SXN-156	104129	0.763226383	1.094222166
		SXN-78	122602	0.766202835	1.098489445
	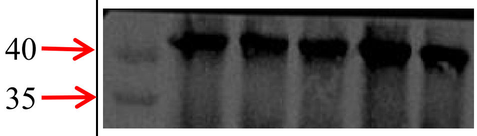	control	82058	1.218284628	1
		model	83843	1.176091027	0.965366385
		EPO	82014	1.417197064	1.163272548
		SXN-156	74532	1.436550743	1.179158557
		SXN-78	70620	1.419668649	1.16530129

Immunoblot images of perilipin-1 in bone marrow cells of each group mice.

Name of protein	WB figures	Group	IntDen
perilipin-1	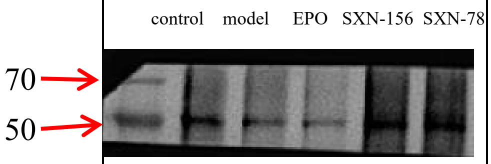	control	80870
		model	56779
		EPO	40348
		SXN-156	112355
		SXN-78	108635
	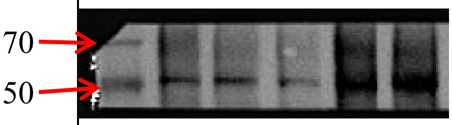	control	54360
		model	49631
		EPO	31936
		SXN-156	98357
		SXN-78	109718
	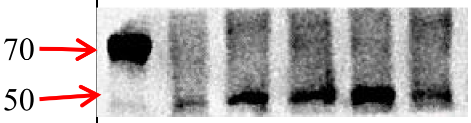	control	59226
		model	90319
		EPO	105852
		SXN-156	118482
		SXN-78	97496

Name of protein	WB figures	Group	IntDen	Intensity ratio	Normalization
ACTIN	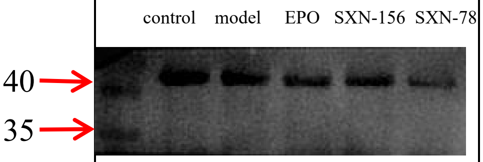	control	109742	0.736910208	1
		model	87794	0.646729845	0.877623675
		EPO	54043	0.746590678	1.013136567
		SXN-156	67329	1.668746008	2.264517429
		SXN-78	41878	2.594082812	3.520215605
	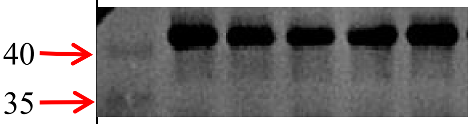	control	126078	0.431161662	1
		model	119574	0.415065148	0.962667103
		EPO	98293	0.324906148	0.753559921
		SXN-156	116329	0.845507139	1.96099796
		SXN-78	132980	0.825071439	1.913601121
	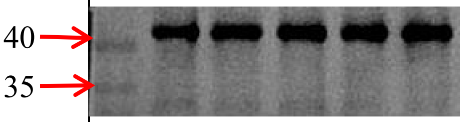	control	78267	0.75671739	1
		model	110774	0.815344756	1.077475906
		EPO	108782	0.973065397	1.28590331
		SXN-156	108029	1.096761055	1.44936679
		SXN-78	116085	0.839867339	1.109882433
